# A Y-shape connection device for pediatric patients with an indwelling catheter (Dia = 8Fr) during urodynamic studies, especially for filling phase measurements: a single-center prospective study for safety and effectiveness

**DOI:** 10.3389/fped.2023.1271417

**Published:** 2023-11-10

**Authors:** Shi-Qin Yang, Xiao Zeng, Liao Peng, Si-Hong Shen, Jie Zhang, Zhi-Hui Huang, Hong Shen, De-yi Luo, Tao Jin

**Affiliations:** ^1^ Department of Urology, Institute of Urology, West China Hospital, Sichuan University, Chengdu, Sichuan, China; ^2^ Pelvic Floor Diseases Center, West China Tianfu Hospital, Sichuan University, Chengdu, Sichuan, China; ^3^ Operating Room, West China Hospital, Sichuan University/West China School of Nursing, Sichuan University, Chengdu, Sichuan, China

**Keywords:** urodynamic study, Y-shape connection device, indwelling catheter, pediatrics, filling phase measurements

## Abstract

**Introduction:**

This prospective study aimed to assess the effectiveness of a Y-shape connection device in reducing pain and bleeding in pediatric patients with indwelling catheters during urodynamic studies (UDS), while also obtaining effective results in the filling phase.

**Methods:**

A total of 45 pediatric patients with a mean age of 13 years were included, all of whom underwent both a UDS with the Y-shape connection device (Method A) and a standard UDS procedure (Method B).

**Results:**

The Y-shape connection device demonstrated similar overall urodynamic parameters compared to the standard procedure, while also resulting in significantly less bleeding (*P* = 0.006) and lower VAS scores during (1.12 ± 0.58 vs. 3.88 ± 1.01, *P* = 0.001) and after (0.12 ± 0.08 vs 2.91 ± 0.89, *P* = 0.001) the procedure. No adverse events were reported at the 1-month follow-up.

**Discussion:**

These findings suggest that the Y-shape connection device can effectively reduce pain and bleeding during and after UDS in pediatric patients with indwelling catheters (Dia = 8Fr), while also obtaining effective results in the filling phase. Therefore, this Y-shape connection device has a more significant value for children who require urodynamic studies and place more emphasis on filling phase parameters.

**Clinical trial registration:**

ChiCTR2300068280.

## 1. Introduction

Urodynamic studies (UDS) are reliable for assessing bladder and lower urinary tract (LUT) symptoms based on hydrodynamics and electrophysiology (1). UDS is widely used in urological diseases, especially in differentiating and diagnosing enuresis nocturia and neuropathic bladder caused by spinal meningocele and rachischisis (2). The classical UDS is well recognized as an invasive procedure in which the measurement catheter must be inserted into the bladder through the urethra to evaluate vesical pressure accurately (3). Most measurement catheters comprise medical polymer plastics with rigidity and characteristics similar to urinary tissue. The catheterization process might damage the urethra or bladder mucosa, leading to bleeding and plugging, particularly in patients with bladder outlet obstruction (BOO) and urethral stricture (4).

For patients with an indwelling catheter (e.g., neurogenic bladder, urinary retention, or post-operation), the medical staff must first remove the indwelling catheter, then put the urodynamic measurement catheter into the bladder via the urethra, and re-catheterize the patient after the procedure, thus undoubtedly increasing the risk of urethral damage, pain, urinary tract infections (UTIs) and decreasing patient satisfaction. It is well known that children are more sensitive to pain than adults, and the pain derived from this complicated process indeed leads to lower coordination and poor quality of test results, especially in children (3). Thus, looking for a method or equipment is imperative to obtain accurate, reliable clinical data and simultaneously avoid re-catheterization in these patients.

Based on the current understanding of urodynamic mechanisms and the relationship between urethral resistance, in our routine urodynamic studies, we typically use a measurement catheter with a diameter of 6Fr. However, in our routine clinical work, the minimum catheter diameter for catheterization is 8Fr, which differs from the 6Fr measurement catheter diameter commonly used during urodynamic studies. Although the diameter of the catheter may increase urethral resistance and potentially affect the measured parameters during the voiding phase, it may not have a significant impact on the measured parameters during the storage phase. Therefore, we directly utilized the patients' indwelling catheter (Dia = 8Fr) with a Y-shape connection device *in vitro* to connect the pump pipe and pressure transmission tube. As we mentioned before, we use the minimum diameter catheter (Dia = 8Fr), but it may also affect the resistant relationship during the voiding phase so that we may pay more attention to evaluate the filling phase parameters. The filling phase parameters include the maximum bladder test capacity (MCC), bladder feeling (BF), bladder compliance (BC), and detrusor overactive contraction (DO). These parameters are critical for us to explain the patients' storage symptoms. The purposes of this study were to introduce the Y-shape connection device and to evaluate whether it could relieve pain and discomfort, as well as we could obtain effective results, especially in the filling phase.

## 2. Methods and materials

### 2.1. Patients selection

**Figure 1:**
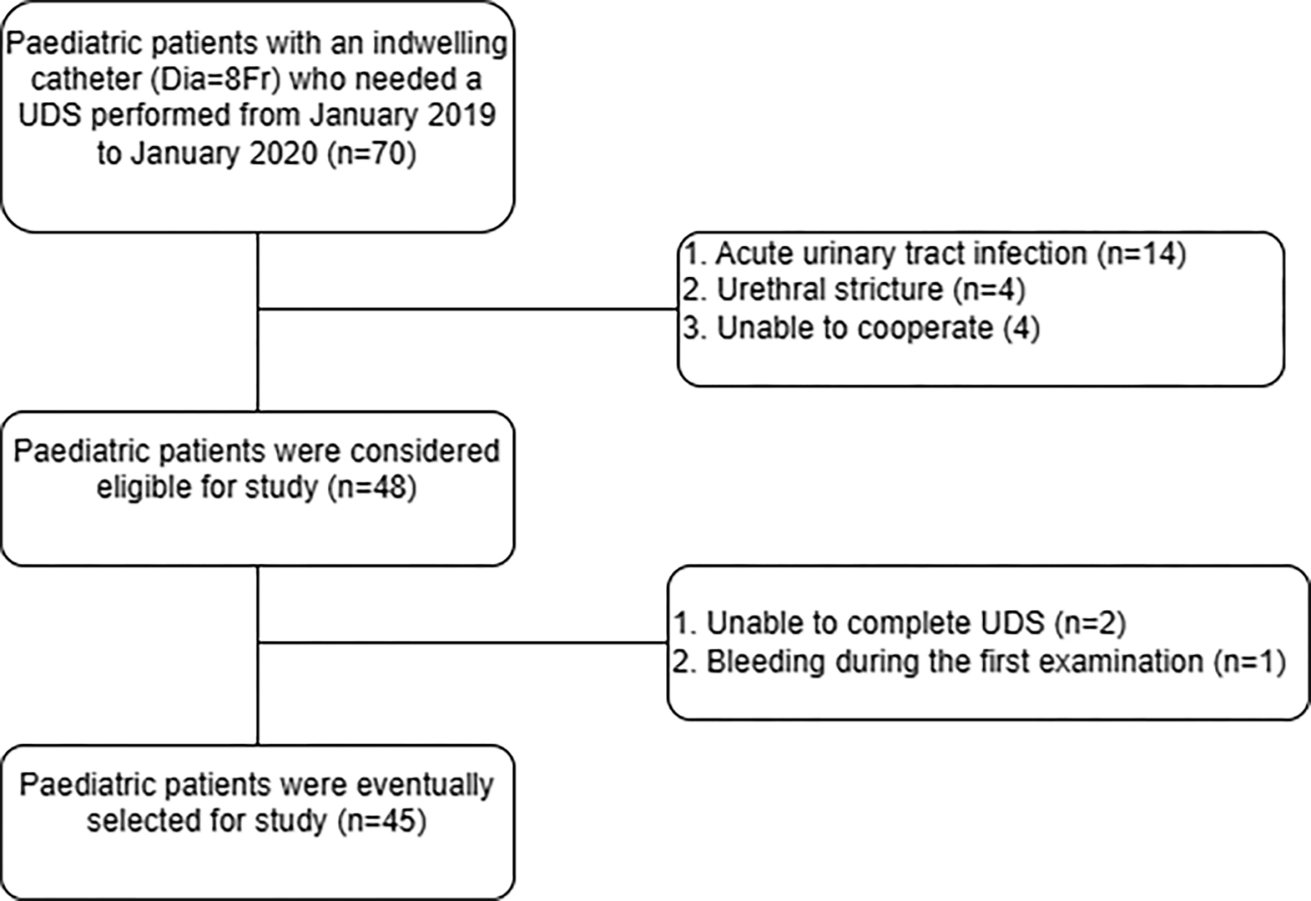
Flow chart of screening patients for UDS.

The study was a prospective study. We included pediatric patients with an indwelling catheter who ran a UDS from January 2019 to January 2020 in the urodynamic center of West China Hospital. Children less than 18 years old with indwelling catheters (Dia = 8Fr) were included in this study. However, patients were excluded according to the following criteria: (1) acute urinary tract infection, (2) urethral stricture (3) patients with an indwelling catheter (Dia = 8Fr) bleed during UDS. The baseline information of selected patients is shown in Table 1. After obtaining ethical approval (#2019187) from West China Hospital of Si Chuan university for this study, patients and their guardians were contacted and subsequently volunteered to undergo follow-up (Figure 1).

**Table 1:** Baseline information of selected children.

Parameters		*N*	%
Age (mean ± SD)	(1) Male	13 ± 2.36	
	(2) Female	13 ± 2.35	
Gender	(1) Male	9	20
	(2) Female	36	80
Diseases	(1) Urinary retension	21	46.7
	(2) Neurogenic bladder	17	37.7
	(3) Others require UDS	7	15.6
Retention time of indewelling cathter (mean ± SD) (days)	(1) Male	3.68 ± 1.93	
	(2) Female	3.68 ± 1.93	

### 2.2. Y-shape connection device

**Figure 2:**
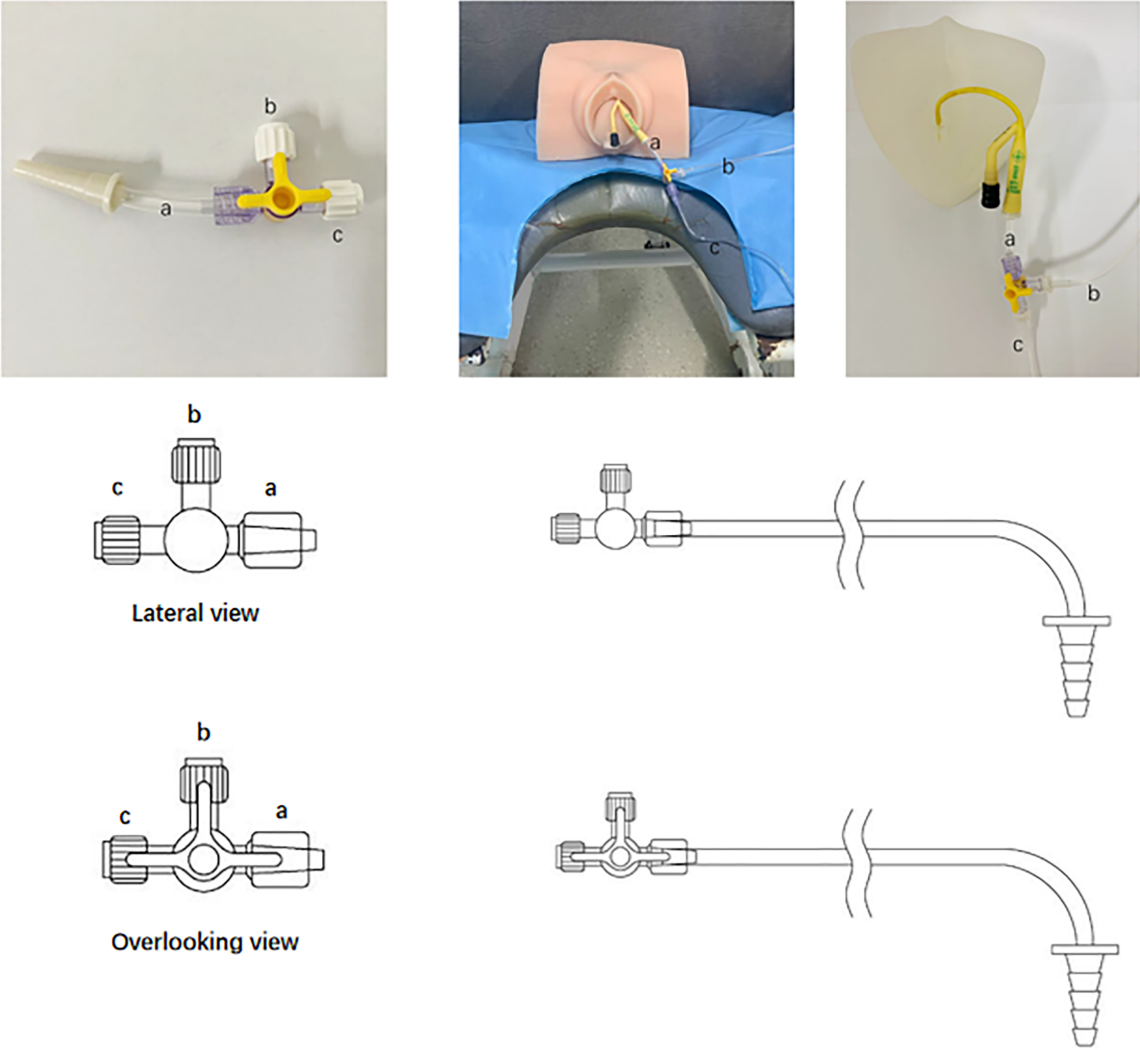
The Y-shape connection device for paediatrics with indwelling catheter during urodynamic test. (**A**) a is a common channel for vesical pressure measurement (**B**) and liquid filling (**C**). (**B**) connected to bladder pressure tube, and (**C**) linked water pump pipe.

We have acquired a patent issued by the National Patent Office of China (NO. 201820007769. X) for the Y-shape connection device currently used in our clinical practice. The Y-shape connection device is a three-way tube with three connectors (Figure 2). All valves are open during the UDS working process. The first connector (Figure 2A) is a common channel for introducing liquids and measuring intravesical pressure; the second connector (Figure 2B) is connected to the bladder pressure measurement tube, and the third connector (Figure 2C) is linked to the water pump tube. We employed the patients' indwelling catheter as a channel to connect the pump tube and pressure measurement tube with the Y-shape connection device to avoid re-catheterization.

### 2.3. Urodynamic study

The measurements and evaluation of urodynamic parameters were performed by the International Continence Society (ICS) guidelines (1) by the same experienced urodynamicist, and all patients provided written informed consent. The air in the measurement tube was removed clearly, and no bubbles remained to avoid signal attenuation in different media. Besides, to prevent the influence of filling and pressure measurement on the results of the same channel, we stopped the perfusion pump and recorded the pressure value (5). Urodynamic machine from Laborie.Co Canada, Triton with water filling system were used in this study. All included children were in the sitting position underwent UDS twice during the same test process with a half-hour interval between examinations to avoid the impact of the previous UDS. In addition, we usually use symphysis pubis as the standard manometry level (7).

Patients underwent UDS with the Y-shape connection device first because the patient's tube conditions would not affect the process. For the Y-shape connection device approach, we should remove the fluid in the water capsule of the indwelling catheter first. We then put the connection device (with ends thread, a) into the opening of the indwelling catheter. Next, we should connect the filling tube and bladder pressure measurement tube with the b and c, then we can run the testing process. Subsequently, a standard procedure was performed (3). In the standard procedure, we should first remove the current indwelling catheter and then put the normal 8Fr water measurement UDS catheter via the urethra into the bladder and another one via rectal into ampullae recti. After all two measurement catheters be placed in the correct position, we could run the UDS process. Data from the two processes were collected and analysed especially data collected in the filling phase). Notably, the infusion medium used in our study was normal saline at room temperature, and the filling rate was 10 ml/min. Importantly, patients with an indwelling catheter were instructed to empty the water sac in the urinary catheter initially to decrease the obstructions caused by the sac (7).

### 2.4. Outcomes

Urodynamic parameters, including maximum flow rate (Qmax), maximum bladder test capacity (MCC), bladder feeling, and the bladder contraction index (PdetQmax + 5Qmax) were collected to confirm the clinical efficacy of the Y-shape connection device (8, 9). Adverse events such as bleeding and plugging occurred during and after the UDS (within 1 month) were recorded.

### 2.5. Statistical analysis

Statistical analysis was performed using SPSS v. 25 software (SPSS Inc. IBM Corp, Somers, NY, USA). Continuous variables are presented as the mean ± SD (standard deviation) or median (range), and differences between methods were tested using Student's *t*-test or the Mann–Whitney *U* test according to the normal or non-normal distribution of the data, respectively; categorical parameters were presented as numbers (percentage), and the chi-square test or Fisher's exact test was applied. A *P* value of <0.05 was considered statistically significant.

## 3. Results

Forty-five children (36 girls and 9 boys) with a mean age of 13 (9–16) years old met the inclusion criteria and were included in the study. Notably, method A had similar performance in the overall urodynamic parameters as the classical UDS approach, and there were no statistically significant differences in Qmax, MCC, bladder feeling, or PdetQmax + 5Qmax (*P* > 0.05). However, method A reported lower VAS scores both during (1.12 ± 0.58 vs. 3.88 ± 1.01, *P* = 0.001) and after the UDS (0.12 ± 0.08 vs. 2.91 ± 0.89, *P* = 0.001) (Table 2).

**Table 2:** Urodynamic parameters and VAS in two methods.

Indicators	Method A	Method B	*P*-value
Qmax (ml/S)	12.25 ± 7.05	12.35 ± 7.36	0.965
First desire (ml)	132 (108–190)	134.5 (110–187)	0.779
Strong desire (ml)	273.00 ± 27.05	277.70 ± 28.92	0.599
MCC (ml)	290 (276–439)	305 (272–438)	0.758
PdetQmax + 5Qmax	140 (14–198)	138.5 (108–192)	0.529
VAS (during UDS)	1.12 ± 0.58	3.88 ±* *1.01	0.001
VAS (after UDS)	0.12 ± 0.08	2.91 ± 0.89	0.001
Bladder Compliance	67.00 ± 69.03	68.02 ± 68.53	0.513
Initial intravesical pressure	20.33 ± 6.85	21.23 ± 6.92	0.452
Initial abdemanal pressure	19.58 ± 7.06	19.27 ± 7.21	0.623

Qmax, peak flow rate; MCC, maximum apacity; VAS, visual analogue scale; Pdet, pressure of detrusor.

Regarding complications, 8 (17.8%) patients experienced bleeding during re-catheterization in the traditional UDS approach. However, 0 patient presented bleeding in method A. Notably, the Y-shape connection device resulted in less bleeding (*P* = 0.006) than the standard procedures. Neither method had plugging or interruption during the UDS. No adverse events were reported at the 1-month follow-up (Table 3).

**Table 3:** Complications during the two procedures (%).

Conplications	Method A	Method B	*P*-value
Plugging	0	0	–
Bleeding	0	8 (17.8%)	0.006
Interruption	0	0	–

## 4. The features of urodynamic study images

### 4.1. The features of UDS traces obtained by the Y-shape connection device

**Figure 3:**
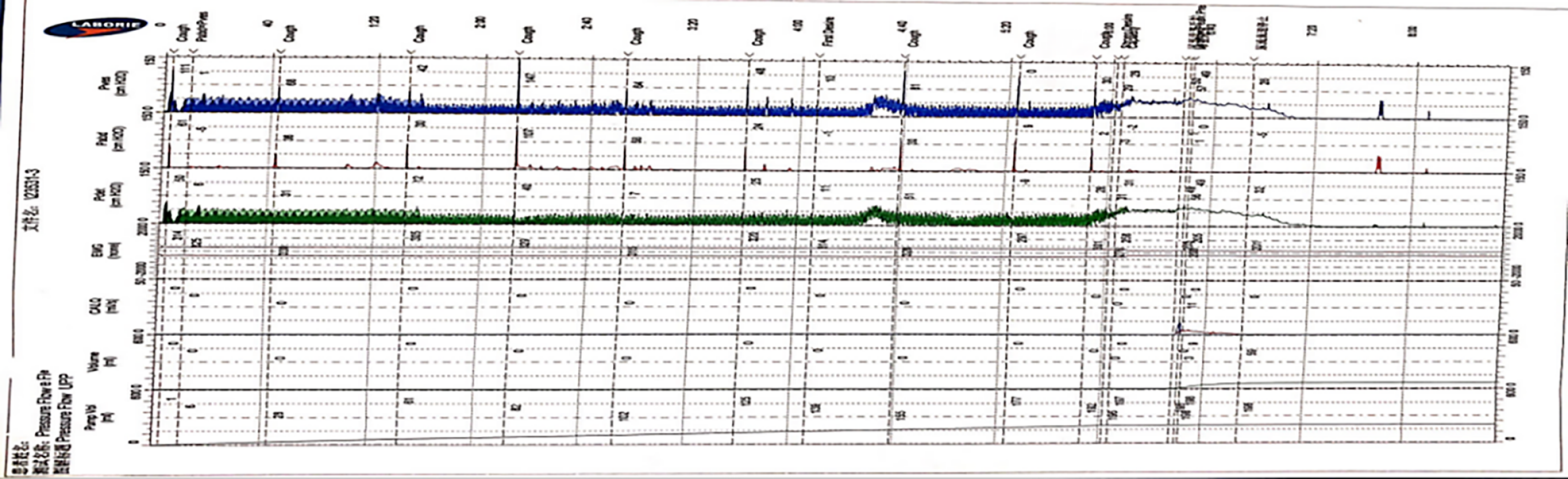
The features of UDS traces obtained by the Y-shape connection device with 12Fr indwelling catheter. (The filling period trace was significantly thickened, and the detrusor overactive contraction waveform was recorded during the filling period, the cough test is not easily distinguished, and the urination period curve became thinner and presented as high detrusor pressure with low flow rate).

**Figure 4:**
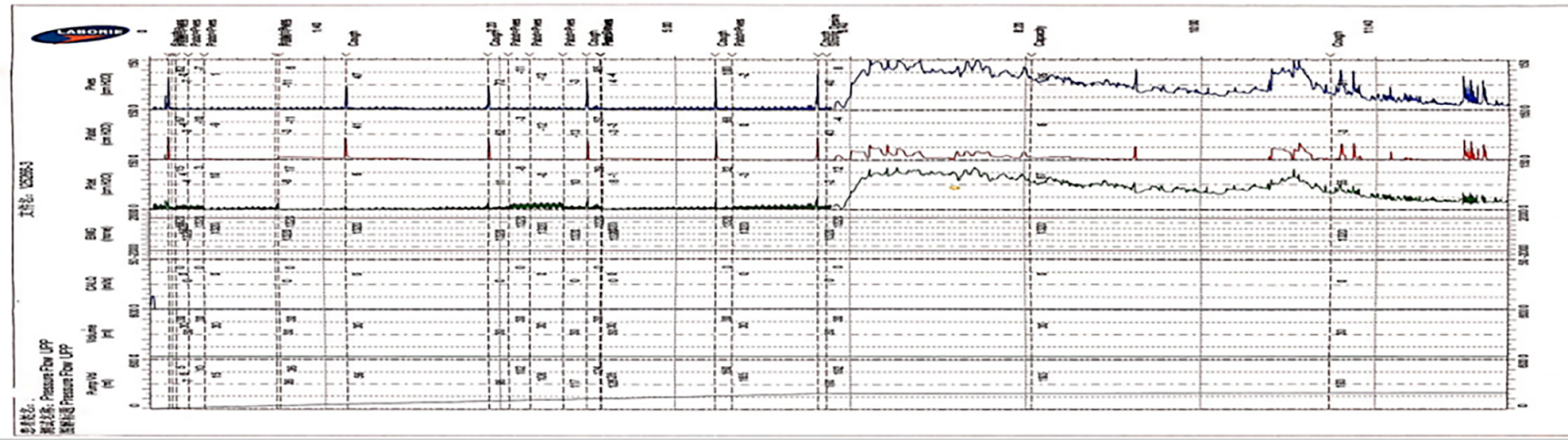
The features of UDS traces obtained by the Y-shape connection device with 8Fr indwelling catheter. (The trace obtained by the Y-shape connection device with 8Fr catheter was slightly thickened than 12Fr catheter, cough test can be clearly recorded, and the urination stage was presented high detrusor pressure with low flow rate).

Compared with the traditional traces, the trace curve obtained by the Y-shape connection device is thicker, and the curve fluctuation is more obvious. Besides, the curve will become thins when the detrusor has inhibitory contractions or during the urination phase. But “Bladder outlet obstruction” is frequently recorded during urination, showing high detrusor pressure and decreased low flow rate in almost all cases (Figures 3, 4).

### 4.2. The features of UDS traces obtained by classical approach

**Figure 5:**
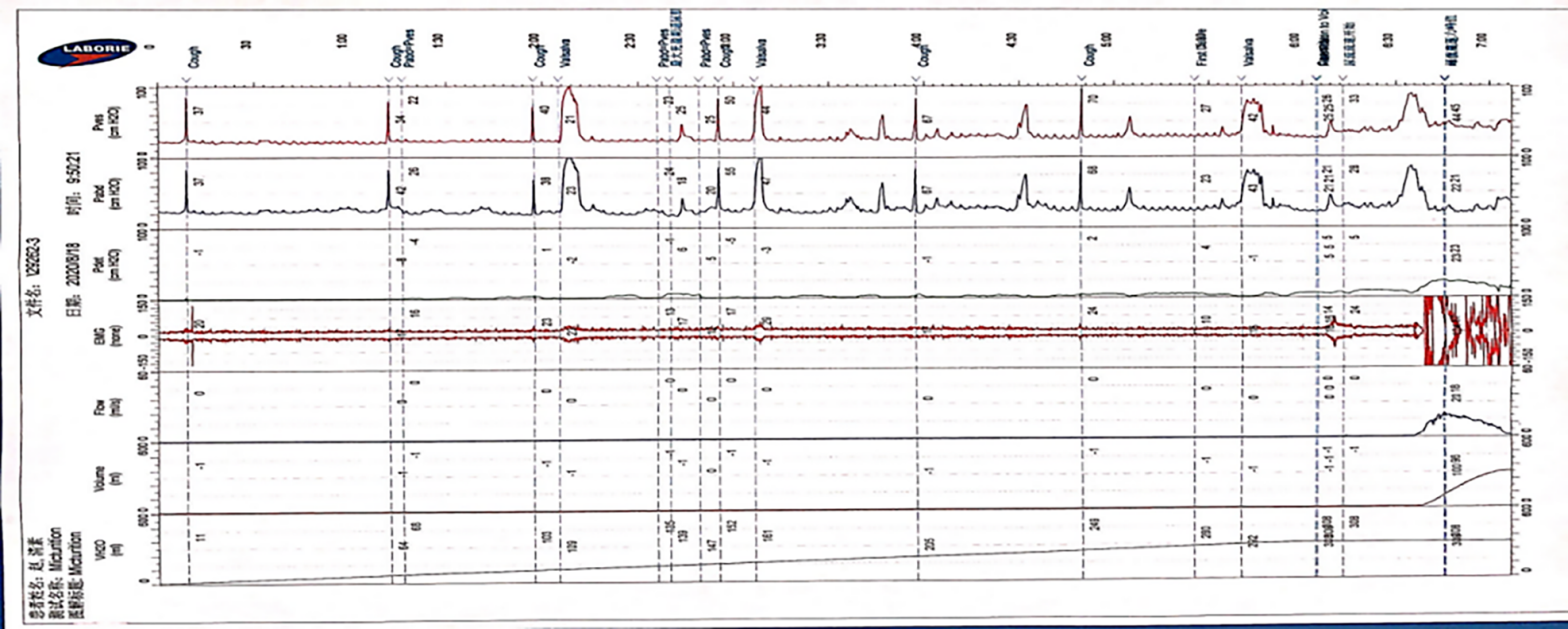
The features of UDS traces obtained by classical approach. (The trace obtained by the traditional approach is smooth and stable, which can clearly record the cough experiment and other UDS parameters, and the urination period can reflect the patient's real urethral resistance relationship).

The traces curve obtained by the classical method is smooth and thin, which can clearly record the waveform of the cough test, which is basically consistent with the traces obtained by connecting the 8Fr indwelling catheter with the Y-shape connection device. We need to emphasize that the traces obtained by the classical method during the urination period can reflect the urethral resistance more authentic (Figure 5).

## 5. Discussion

The use of UDS is limited in clinical practice for pediatric urology because of the invasiveness of the current process, which may increase the risk of damage to the tissue and lessen patient satisfaction, especially in high-risk patients, such as the youngest or those with underlying comorbidities. Bleeding and pain are often unavoidable in the insertion process, which might lead to a UTI and plugging after the UDS, particularly in patients with indwelling catheters and after a long period of follow-up (10). The best method for performing a standard urodynamic test in patients with indwelling catheters has been an ongoing issue in our clinical practice. Given that UDS is a powerful tool in the pediatric department of urology, it is imperative to look for a method or equipment to obtain accurate, reliable clinical data and simultaneously avoid re-catheterization in these selected patients. The connector developed at our institute undoubtedly simplifies the UDS process.

The Y-shape connection device had comparable performance in overall urodynamic parameters compared with classical UDS; however, the Y-shape connection device resulted in lower VAS scores both during (1.12 ± 0.58 vs. 3.88 ± 1.01, *P* = 0.001) and after the UDS (0.12 ± 0.08 vs. 2.91 ± 0.89, *P* = 0.001). Regarding complications, the Y-shape connection device resulted in significantly less bleeding (*P* = 0.006). No plugging or interruption occurred during the UDS in either method. It is worth mentioning that although we used “bleeding” during UDS as an exclusion criterion, only one of the 46 patients we first collected had bleeding. Considering the possible adverse effects (bleeding, plugging) of re-performing traditional urodynamic testing, we excluded the patient, but it was not difficult to find that Y-shape connection device has critical value in reducing bleeding.

Notably, the current UDS process for comparison with the procedure that employed the Y-shape connection device remained the same. Additionally, a 6-Fr urinary tube may not affect the measurements and accuracies in the voiding period (11). But for our current study, we use the 8Fr catheter to run this process, so one thing we want to emphasize is that we cannot ignore the influence of the urethra-resistant relationship during the voiding phase, so for our current study, we may pay more attention to the storage phase parameters instead of voiding phase parameters. Emptying the catheter water sac in the urinary catheter may decrease the obstructions caused by it. Therefore, it was unsurprising that the Y-shape connection device presented the same diagnostic effect as the standard UDS. The similar urodynamic outcomes in the two methods confirmed our notion and further verified its efficacy.

Most children who needed take a UDS had relatively severe LUT symptoms, such as leaks or dysuria. Long-term LUT symptoms can easily lead to a UTI, inflammation, and tissue oedema (9); consequently, high sensitivity to pain or bleeding occurs more often in these selected patients. The Y-shape connection device avoided re-catheterization, which directly reduced the pain and improved the children's compliance during the UDS, especially for diseases that need UDS evidence to explain the storage symptoms, like urinary urgency, urinary frequency, and incontinence (urgency incontinence).

Because UDS is an invasive procedure, artifacts may influence the accurate interpretation of results (12). Despite all efforts to achieve normalcy, the test environment is unnatural; most children are apprehensive to the degree that can affect the findings. To reduce anxiety, the study was best performed with the child seated, watching cartoons and accompanied by their parents. Only essential equipment should remain in the room. Avoiding general anesthesia is crucial because it affects the natural state and eliminates the chance of voiding (13). Intranasal midazolam may be administered in certain situations where high anxiety levels cannot be mollified, as this drug appears innocuous in terms of its effect on the outcome of the study (14).

Comparing the traces obtained by the traditional approach and the Y-shape connection device, we can observe that the trace curve obtained by the Y-shape connection device is relatively thick, and it exhibits slight fluctuations in the overall trend of the trace. In the urination stage, the trace recorded with the Y-shape connection device predominantly demonstrates increased detrusor pressure alongside a decreased flow rate (BOO feature). We also compared the results of the different diameters indwelling catheter connected with the Y-shape connection device, and we found that the trace obtained by the 8Fr indwelling catheter with the Y-shape connection device was finer and more similar to the trace obtained by the traditional approach than that of the 12Fr indwelling catheter. The reasons for the thickening and fluctuation of the curve were analyzed, which may be caused by the fluid flow in the lumen caused by the diameter of the urinal tube. Therefore, considering that the diameter of the 8Fr indwelling catheter is closest to the traditional 6Fr catheter used in urodynamic studies, and based on the measurement results obtained with different catheter diameters, choosing 8Fr is currently the best option. Up to now, the traditional pressure measuring tube commonly used material is polyvinyl chloride, and the material of catheter is often silicagel; there is currently no clear literature detailing the specific effect of the material of the pressure tube on the measurement results. Based on our measurement traces, we found that the trace quality does not affect the parameter acquisition and result interpretation in the 8Fr diameter catheter. For these reasons, the Y-device has a similar effect to that of traditional devices for measuring the storage period, which coincides with the more concentrated storage period symptoms of the neurogenic bladder, but it has an essential value in reducing bleeding and pain during UDS.

## 6. Study limitations

The first limitation of the study is that the sample size in the current article was relatively small; thus, a trial with a larger sample size should be an aim in the future. Also, the existing Y-shape connection device is a promising alternative to the standard UDS approach for kids with indwelling catheters (8Fr). Due to the current knowledge of the urethral resistant relationship, only diameter < 6Fr may not significantly influence, even though our study uses the minimum diameter indwelling catheter (8Fr). Still, we may need further investigation to know whether this diameter will significantly influence the voiding phase and the urethra-resistant relationship, so our study can only predict the effectiveness in the filling phase (storage phase) subject of ongoing research.

## 7. Conclusion

The Y-shape connection device results in less pain and bleeding during and after the UDS and is a promising alternative for children with an indwelling catheter for who require urodynamic studies and place more emphasis on filling phase parameters, especially for children with neurogenic bladder.

## Data Availability

The raw data supporting the conclusions of this article will be made available by the authors, without undue reservation.
